# Droplet-based lab-on-chip platform integrated with laser ablated graphene heaters to synthesize gold nanoparticles for electrochemical sensing and fuel cell applications

**DOI:** 10.1038/s41598-021-88068-z

**Published:** 2021-05-07

**Authors:** Sangam Srikanth, Sohan Dudala, U. S. Jayapiriya, J. Murali Mohan, Sushil Raut, Satish Kumar Dubey, Idaku Ishii, Arshad Javed, Sanket Goel

**Affiliations:** 1 Department of Mechanical Engineering, Birla Institute of Technology and Science, Hyderabad, 500078 India; 2 MEMS, Microfluidics and Nanoelectronics Laboratory, Department of Electrical and Electronics Engineering, Birla Institute of Technology and Science (BITS) Pilani, Hyderabad Campus, Hyderabad, 500078 India; 3 Digital Monozukuri (Manufacturing) Education Research Centre, Hiroshima University, Higashi-Hiroshima, Hiroshima 739-0046 Japan; 4 Smart Robotics Lab, Graduate School of Engineering, Hiroshima University, Higashi-Hiroshima, Hiroshima 739-8527 Japan

**Keywords:** Electrical and electronic engineering, Mechanical engineering

## Abstract

Controlled, stable and uniform temperature environment with quick response are crucial needs for many lab-on-chip (LOC) applications requiring thermal management. Laser Induced Graphene (LIG) heater is one such mechanism capable of maintaining a wide range of steady state temperature. LIG heaters are thin, flexible, and inexpensive and can be fabricated easily in different geometric configurations. In this perspective, herein, the electro-thermal performance of the LIG heater has been examined for different laser power values and scanning speeds. The experimented laser ablated patterns exhibited varying electrical conductivity corresponding to different combinations of power and speed of the laser. The conductivity of the pattern can be tailored by tuning the parameters which exhibit, a wide range of temperatures making them suitable for diverse lab-on-chip applications. A maximum temperature of 589 °C was observed for a combination of 15% laser power and 5.5% scanning speed. A LOC platform was realized by integrating the developed LIG heaters with a droplet-based microfluidic device. The performance of this LOC platform was analyzed for effective use of LIG heaters to synthesize Gold nanoparticles (GNP). Finally, the functionality of the synthesized GNPs was validated by utilizing them as catalyst in enzymatic glucose biofuel cell and in electrochemical applications.

## Introduction

Integration of various systems and processes on a microfluidic platform enables the development of lab-on-chip (LOC) devices, which have proven applications in a variety of biomedical and biochemical domains^1,2^. Further, temperature control in such devices is necessary for LOC applications such as microfluidic PCR^3^, Electrophoresis^4^, Digital microfluidics^5^, mixing of fluids^6^ and protein crystallization^7^ etc. In addition, applications involving droplet microfluidics such as colloidal molecules preparation^8^, droplet based PCR devices^9^ and droplet based material synthesis^10,11^ have a great need of thermal management for realizing final outcomes. Also, controlled temperature is extremely critical in droplet microfluidics as the droplet size and shape varies rapidly even with smaller changes in the temperature^12^.

During the last decade, many micro-heating techniques have been proposed such as Peltier and Joule heating^13,14^, heating through chemical reactions^15^, lasers and microwaves^16,17^, pre-heated fluids etc. The temperature ranges offered by these techniques vary from 5 to 130 °C^18^. Recently, heaters based on Indium Titanium Oxide (ITO) have become popular and are commercially available because of their transparency and high electrical conductivity^19^. However, traits like delayed response, complex manufacturing process, high cost due to scarceness of indium and fragile nature, limit its application in microfluidic and lab on chip devices. Literature also highlights heaters fabricated using silver nanoparticles^20^ and metal wires such as silver nanowires^21^ and copper nanowires^22^. Gold based Nano heaters^23^ have also been described in the literature. Nonetheless, the aforementioned heaters, though popular, are less durable, expensive, rigid, high power consuming, and have complex manufacturing and post processing. For peltier and joule heating, high temperature gradient, high response time and bulky system make it difficult to realize an integrated microfluidic device. With reference to microwave heating, additional equipment such as signal generator, power amplifier waveform generator and a DC power supply as well makes the entire system huge and difficult to integrate with microfluidic device^24^. In case of nanowire heating, the fabrication includes spray deposition of nanoparticles on to the substrate which requires post processing techniques^21^ and in addition they get oxidized below a temperature of 100°C^22^ and are costlier as well. The same is true in the case of inkjet based silver nanoparticle coating which requires post processing techniques such as controlled annealing and electrodeposition. It was also reported that these heaters might result in unstable results due to improper annealing^20^. Few works reported the use of gold film as a micro heater which obviously hinders its use owing to high cost^25^. Some of the available parameters of existing heating technologies employed in microfluidics are tabulated (Table 1).

**Table 1:** Few Existing heating techniques employed in Microfluidics.

Heating method	Temp. Range (°C)	Accuracy (°C)	Response time	Power (mW)	Cost	Fabrication tech/mat
Preheated Liquids through Peltier^26,27^	5–100	$\pm$ 0.1	3–5 min	1000–3000	Low	Cartridge heating element
IR/laser heating^28,29^	25–1500	$\pm$ 1 °C	5–30 s	50–500	Medium	IR Lamp
Joule heating^30,31^	25–100	$\pm$ 3–5 °C	5-10 s	200–800	Medium	Resistive heating
Microwave^24,32^	28–80	$\pm$ 4 °C	1 s	500–1000	Medium	Microwave resonator
ITO heaters^33,34^	25- 145	$\pm$ 1 °C	30–60 s	500–3000	High	ITO coating
Silver/Gold NP heater^20,25^	25- 300	$\pm$ 1–2 °C	3 s	1000–1700	High	Inkjet printed
Metal nanowires^21,22^	50–175	$\pm$ 4–7 °C	1-5 s	1000–4000	High	Spray deposition
LIG (this work)	25–589	$\pm$ 5 °C	10 s	500–1000	Low	Laser ablation

The present work focuses on deterministic control and monitoring of heat flux in droplet-based microfluidic devices which is one of the important functionalities of modern LOC devices. The essential parameters for temperature management of such device are heat flux uniformity, wider temperature ranges, good accuracy, capability of rapid heating and quick switching and cooling. These controlled parameters of thermal management are essential in realizing many biological, chemical and physical applications in droplet based microfluidic platform^18^.

This work reports a thin film heater fabricated through the ablation of a CO_2_ laser over a polyimide sheet. This laser ablation on polyimide sheet was first reported by Lin et al., when laser was scribed over a polyimide sheet^35^. On further investigation, it was identified that the resultant formed was three dimensional graphene and was so called as laser induced graphene (LIG). The structure of graphene was identified to have five and seven membered rings unlike the conventional honeycomb structure^36^. LIG has a crystalline graphene structure with a porous morphology that resulted its usage in different applications such as optoelectronic devices^37^, electrochemical sensors and sensing applications^38,39^, heating devices^40^, enzymatic biofuel cells^41,42^, chemical fuel cells^43^, flexible electronics^44,45^ etc. In addition, owing to their porous nature and chemical resistance, they have been utilized to transport fluids acting as porous microchannel and as flexible electrodes in membrane less microfluidic redox batteries^46,47^. Another distinguished feature of LIG is the ability to exhibit change in resistance in response to weak vibrations. This principle was utilized for developing an artificial throat and also for detection biological activities^48,49^. LIG is also well known for its high capacitance to create a supercapacitor exhibiting high power density and current density while maintaining its flexibility^50,51^. Due to its high surface area, good electrical and thermal conductivity, LIG was also being used as active catalyst in electro catalysis applications^52^. As mentioned, LIG has a porous morphology and provides good electrical conductivity and electro thermal performance. However, the performance of the LIG film can be altered by changing the parameters of laser. By tuning the parameters, the morphology of the surface can be varied by changing the intensity of irradiation from sheet to fiber to droplets^53^. Also, the surface of the LIG can be modified to hydrophilic with a contact angle of 0° to a super hydrophobic surface with a contact angle more than 150° based on the application^36,54^. Though, LIG was formed on polyimide sheets, LIG can also be developed on substrates such as cloth, paper, natural coal, Kevlar and also on food for flexible/biodegradable electronics^55^. This simple process gives a promising way for fabrication of large scale flexible material that can be used in diverse applications.

For appropriate utilization in all these applications, LIG is known well for its electro-thermal performance. When a potential is applied over the edges of the developed LIG, thermal energy is generated all over the film. Flexible heaters can reach a very high steady state temperature quickly and can be fabricated using this simple process^56,57^. In addition, through repeated laser ablation, better electro-thermal performance can be achieved for LIG based heaters^56^. Also, LIG patterns with temperature gradients can be obtained by varying the laser parameters for manufacturing of composites^40^. Compared to the above mentioned heaters, the LIG heaters are well known because of (i) ease and environment friendly fabrication process that does not include any post processing techniques such as chemical treatment or high temperature reaction conditions, (ii) the design can be custom made based on the application, (iii) large scale fabrication of LIG films can be achieved at relatively very less cost, (iv) they are thin and flexible, (v) wide range of applications. These advantages of LIG provide a trustworthy option for their usage as heater for microfluidic applications.

Leveraging such advantages, in this work, LIG heaters were fabricated for different combinations of the laser power and the scanning speed of the laser. Although, few works have been reported to study the electro-thermal performance of LIG heater with respect to the variation in laser power yet, the effect of scanning speed has not been studied in detail. The structural, electrical and thermal performances of the heaters were comprehended for different combinations of power and speed in the present work. The LIG heaters with optimal combination of power and scanning speed of the laser was identified based on electrical and thermal performance. Subsequently, the identified LIG was integrated with droplet based microfluidic system making it amenable for diverse lab on chip needs such chemical, biochemical and genomic assays as well as point of care diagnostic applications. The present work demonstrates the utilization of the integrated platform for the synthesis of GNPs in a droplet based microfluidic platform at controlled temperature. Finally, the synthesized nanoparticles were tested in enzymatic bio fuel cell and electrochemical applications.

## Results and discussions

In this section, different combinations of power and speed of laser for fabricating the conductive film and their structural and chemical characterizations are discussed. Also, the thermal and electrical properties of the films are discussed followed by GNP synthesis using droplet-based microfluidics integrated with the fabricated heater. The synthesized GNP were also corroborated for functionality by utilizing them as catalyst in enzymatic glucose biofuel cell (EBFC) and in electrochemical detection of analytes such as uric acid and dopamine.

### Combination of power and speed of laser for fabrication of conductive films

In order to fabricate the conductive patterns, various combinations of laser power and scanning speed were considered. The maximum power of the CO_2_ laser machine was 30 W and the scanning speed was 250 mm/s. The power values were varied from 2.5 to 15% (0.75 W to 4.5 W) in an increment of 2.5% (0.75 W). The scanning speeds were selected in such a way that at a combination of power and speed, the conductive pattern was formed starting with minimum speed and to a point where the pattern was ruptured due to the increase in the scanning speed. For combinations with lesser laser power, the carbonization process was not initiated. Whereas for few sets, higher laser power with combination of higher scanning speeds resulted in irregular and powdered morphology of the pattern. The conductivity of such combinations was not detected by using the 4 probe machine. For instance, at 12.5% power, speeds were selected from the minimum value (1.5%) for fabricating the conductive film. However, carbonization was not initiated up to a speed of 3.5% and the film got ruptured beyond speed of 19%. Thus, combinations where carbonization process was uniform (speeds from 4.5 to 19% at 12.5% power) were considered for fabrication. In a similar fashion, the heaters were fabricated for other values of power at different speeds. Overall, with 58 combinations, different patterns were fabricated. Figure S1 shows the variation in carbonization process observed for different combinations of speed and power. The thickness of the patterned LIG film was found to be 50 µm using a contact profilometer.

### Structural characterization

**Figure 1:**
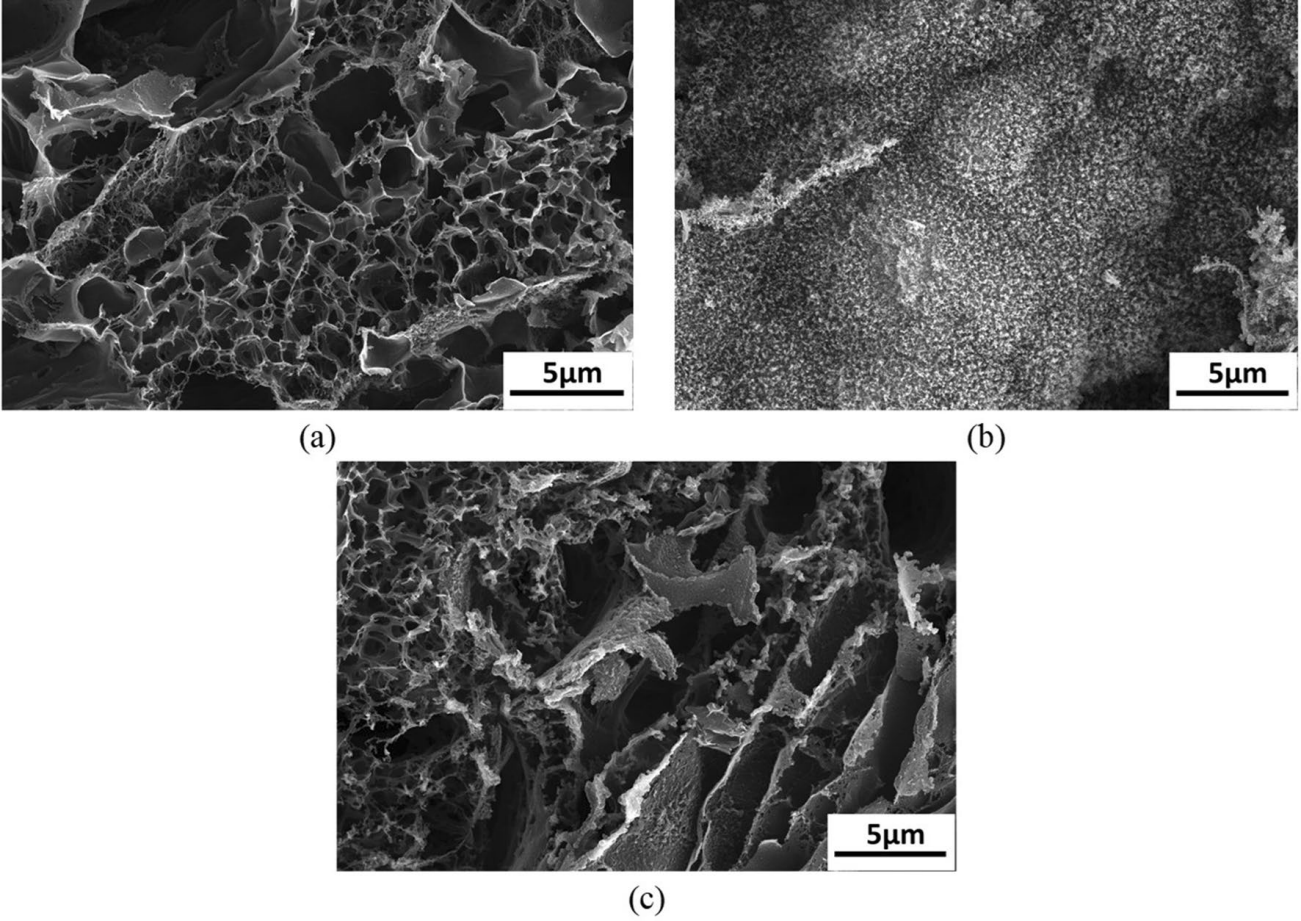
Scaning electron microscopy images of (**a**) porous morphology of the laser ablated pattern (**b**) improper carbonization obtained for few combinations showing the absence of porous morphology (**c**) LIG pattern after subjected to heating for 3 h.

Figure 1 shows the scanning electron microscopy (SEM) image of the fabricated conductive film. Figure 1a shows the film with porous morphology fabricated at a combination of 15% power and 5.5% speed. Figure 1b shows the combination of power and speed where the graphene oxide layer was not formed. Instead, the layer got burnt and powdered ash was observed on the surface of the film that can be eroded by blowing air over it. Figure 1c shows the morphology of the conductive film post heating. The film was subjected to a voltage of 12 V and heated to a temperature of 300 °C and was left for 3 h. SEM images were taken at this stage, which reveal no significant change in the morphology of the fabricated heater. This also indicates that the fabricated heater was capable of bearing heat for longer periods (3 h) while retaining the morphology.

X-ray photo electron spectroscopy (XPS) was performed on the obtained conductive films to understand the chemical changes induced due to the laser ablation. The spectra obtained were calibrated with the carbon peaks of 284.8 eV. Figure S2 shows the XPS spectra obtained for the films with combinations of 15% power and 5.5% scanning speed and 10% power and 15% scanning speed of the laser. The specific combinations were chosen as extreme conductivity values were measured with such combinations. This can also be seen in Figure S2 wherein the carbon percentage obtained in the first combination was found to be 94.1% whereas in the second combination, the carbon percentage reduced to 89%.

### Electrical and thermal characterization

After the fabrication of all the conductive films for different combinations, a 4 probe (Ossila's Four-Point Probe System) machine was used to measure the electrical conductivity of the films. The conductivity was measured to identify the pattern which would give highest thermal energy. As per Eq. (1) (where *P* is power, V is applied voltage and *R* is resistance), a pattern with lowest resistance can transduce high thermal energy for a given electrical energy.

1 $$ P = \frac{{V^{2} }}{R} $$

The films with lower resistance will have higher conductivity values and hence the conductivity values are measured and are shown in Table 2. Though the conductivity values are determined for all the 58 combinations, only the combinations that has exhibited highest and lowest conductivity values are presented in the table.

**Table 2:** Conductivity values of various patterns for the combinations of 15% and 10% power.

**Power of 15%**										
Speed (%)	5.5	6.5		7.5	8.5	9.5	11	12	13	14
Conductivity ($\times$ 10^2^ S/m)	11.29	9.66		8.48	7.97	7.21	6.57	5.49	4.95	4.79
Speed (%)	15	16		17	18	19	20	21	22	23
Conductivity ($\times$ 10^2^ S/m)	4.62	4.52		3.9	3.61	3.21	3.00	2.95	2.82	2.48
**Power of 10%**										
Speed (%)	4.5	5.5	6.5	7.5	8.5	9.5	10.5	11	12	
Conductivity ($\times$ 10^2^ S/m)	8.75	7.58	6.32	4.93	4.13	3.85	3.51	3.34	3.06	
Speed (%)	13	14	15							
Conductivity ($\times$ 10^2^ S/m)	2.74	2.54	1.97							

**Figure 2:**
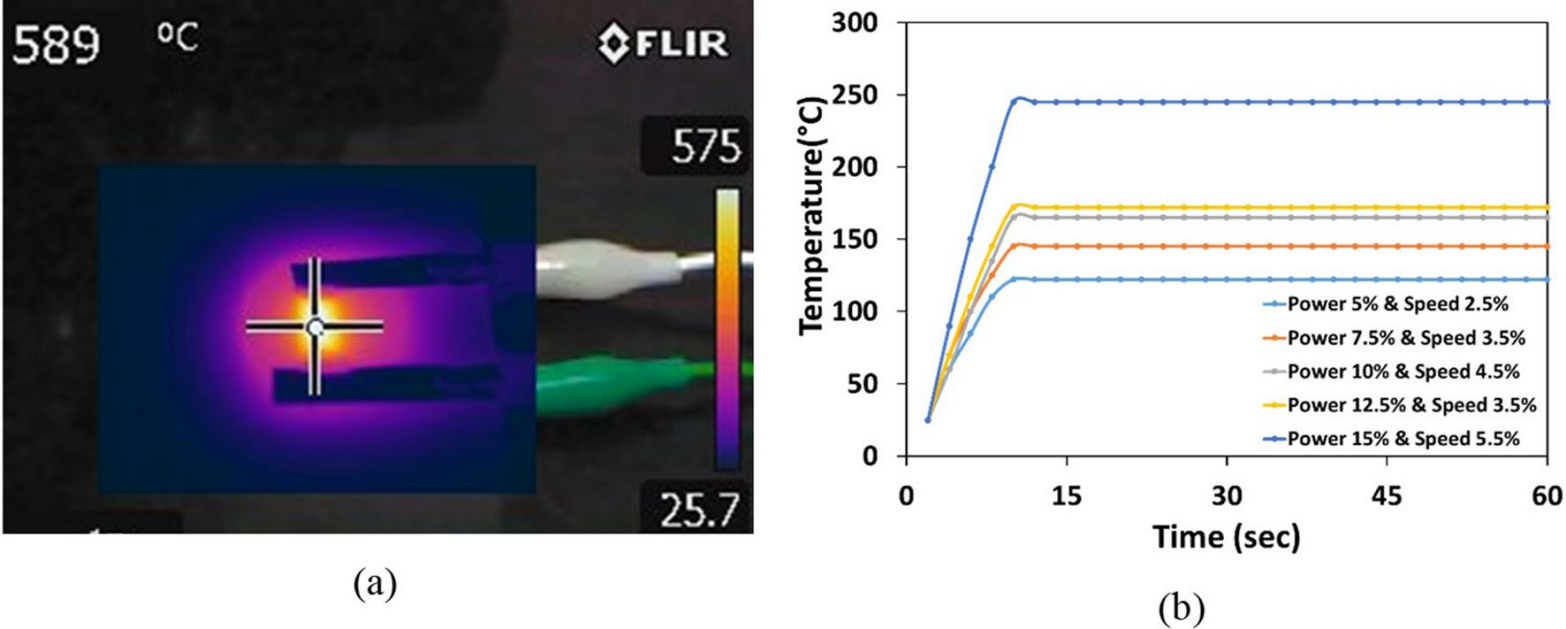
(**a**) Image from thermal camera showing a maximum temperature of 589 °C for an application of 15 V, (**b**) Time versus temperature plot for different combinations of heaters at a voltage of 9 V.

As mentioned, carbonization process was not initiated for speed values below 5.5% with a combination of 15% power and hence was not listed in Table 2. Likewise, as shown in Fig. 1 (b), beyond speed of 14%, the pattern obtained was found to be in powder. To measure the conductivity of the samples, an input voltage of 10 V and a current of 100 mA with an increment of 0.01 V was supplied to the film. These changes in conductivity values implies that with increase in the power of the laser, the resistance value decreases resulting in higher conductivity. Figure S3 (a) shows the linear nature of the graph depicting that the conductivity is uniform over the entire film for both the combinations. Also, the conductivity values of all the fabricated patterns were plotted in Figure S3 (b). It is evident from the figure that an increase in the power of the laser with a combination of lower scanning speed yielded higher conductivity. A minimum value of 1.978 $\times$ 10^2^ S/m was obtained for combination of 10% power and 15% speed and a maximum value of 11.98 $\times$ 10^2^ S/m conductivity was achieved for 15% power and 5.5% speed. Therefore, these two patterns having higher and lower electrical conductivity were chosen for thermal characterization. The key parameters involved in evaluating the heating performance of the film are response time and steady state temperature. Response time is the time taken to reach the steady state temperature which depends on parameters such as morphology of the film, thickness of the sheet, thermal conductivity properties, voltage losses from power source and ambient temperature conditions. In applications involving rapid switching of temperatures, the response time plays a crucial role. However, the steady state temperature is much desired quantity in case of a graphene heater wherein the temperature has to be stable for longer period of time. In this work, a maximum temperature of 589 °C was obtained for an applied voltage of 15 V for a combination of 15% power and 5.5% scanning speed as shown in Fig. 2a*.* However, beyond 15 V, the substrate was unable to resist high temperatures and the graphene oxide layer started rupturing and also the polyimide sheet started burning. The temperatures for different films fabricated at different parameters were calculated and plotted in Fig. 2b wherein time dependency of the temperature can be seen. Also Fig. 2a shows uniform temperature distribution over the entire film captured using the thermal imaging camera. Also, a comparison between the 2 films, i.e., 15% power and 10% power combinations, was performed. It was observed that the combination of 15% power and 5.5% scanning speed showed high thermal performance compared to the other combinations. At a supply voltage of 6.5 V, the combination of 10% and 15% power and scanning speed offered a temperature of 90 °C, whereas the combination of 15% and 5.5% power and scanning speed offered a temperature of 168 °C. In order to comprehend the heating homogeneity, the effective area of the heater was considered for combination of 15% power and 5.5% scanning speed. For an applied voltage of 15 V, the maximum temperature attained was 589 °C with an average temperature 498.91 °C and a standard deviation of 79 °C. However, for heater with a combination of 10% power and 15% and scanning speed, at 6.5 V, the average temperature and the standard deviation were measured and was found to be 81.5 °C and ± 8 °C respectively at a maximum temperature of 90 °C (Figure S4(a)). These values suggest that the homogeneity of the heater at lower temperatures is superior to that of higher temperatures. In addition, to understand the performance of the heater, the current drawn by the heater for a given voltage was also measured and was plotted (Figure S4(b)). It was observed that to obtain a temperature of 90 °C, the heater with 10% power and 15% speed consumes a power of 0.98 W (0.15 A and 6.5 V), whereas the heater with 15% power and 5.5% consumes a power of 0.8 W (0.23 A and 3.5 V).

## Application

### Droplet based microfluidic platform for synthesis of gold nanoparticles

The droplet based microfluidic devices have been reported to be used for synthesizing magnetic iron oxide nanoparticles^58^, silica microspheres^59^, gold nanorods^60^, silver nanoparticles^10^, and gold nanoparticles^11^ using different methods and chemicals. In order to synthesize nanoparticles, temperature is a key factor that effects the size of the nanoparticles. Usage of temperature bath^61^, ceramic heater^62^, heating coils wound to the syringes^60^ and peltiers^63^ and cartridge heaters^64^ for providing temperature were earlier reported to synthesize GNP in a continuous and a droplet based microfluidic platform. However, the above means for supplying temperature to the microfluidic device makes the device bulky and expensive, making it far from the definition of microfluidics. In this work, application of the fabricated thin film graphene heater as a temperature source for synthesis of nanoparticles is performed, which is directly integrated to the microfluidic device.

### Fabrication of droplet based microfluidic device

**Figure 3:**
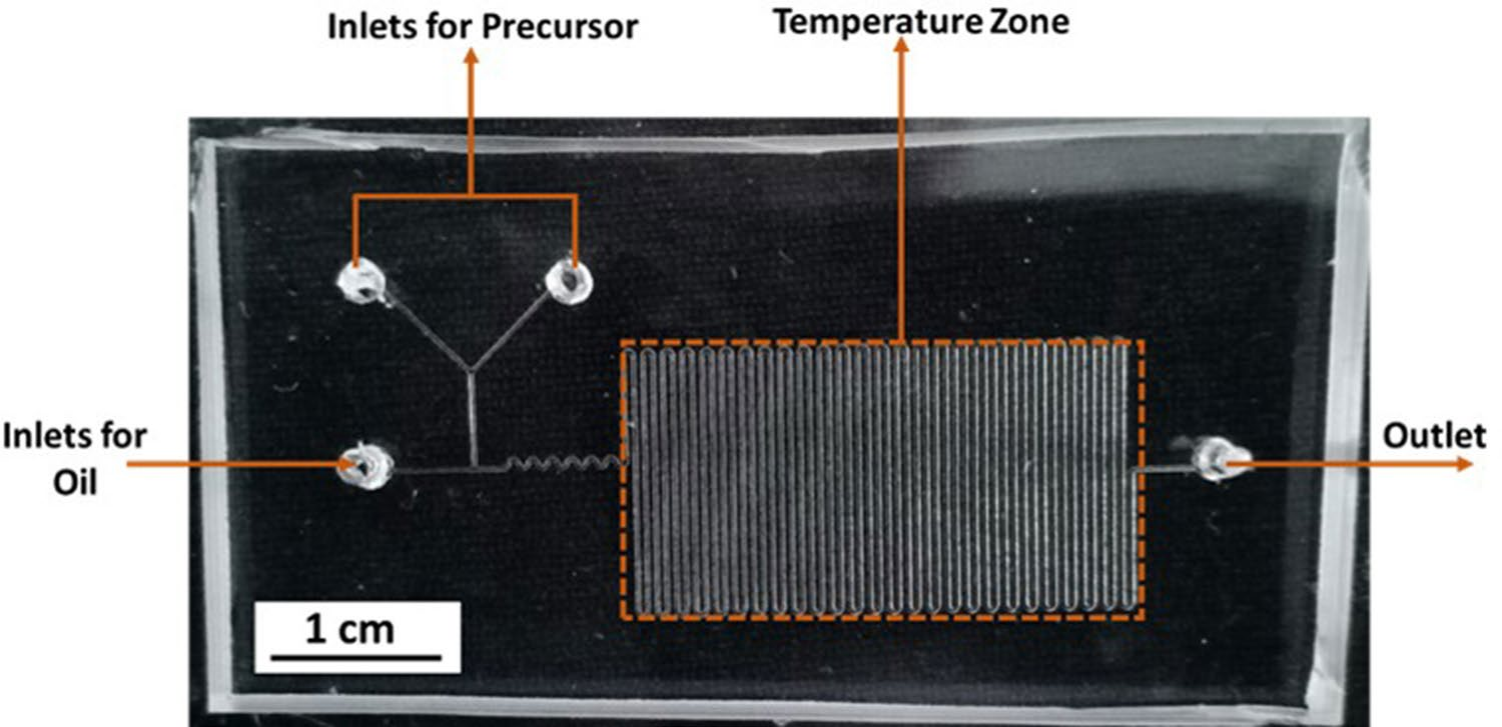
PDMS based droplet microfluidic channel bonded on to a glass substrate. The channel has a uniform width of 175 µm and a depth of 35 µm throughout. The length and breadth of temperature zone is 2.5 × 1.5 cm.

The droplet-based microfluidic device is fabricated as similar to our previous work utilizing direct laser writing^65^. Briefly, a dry film negative photoresist was laminated over a glass substrate using a hot roll laminator. A CAD model of the required channel was designed and was imported into the Direct laser writing machine which converts the ‘.dxf ’file to a G-code. This G-code was transferred to the scanning stage as a tool path. The substrate moves under the laser and the pattern was printed over the photoresist. The pattern consists of a Y-shaped microchannel provided with two inlets for precursors and another inlet for oil to generate droplets. A serpentine channel was provided next to the generation of droplets to ensure proper mixing of the fluids. The heating zone consisted of 50 channels combined as a serpentine as shown in the Fig. 3. The length and width of the temperature zone are 2.5 × 1.5 cm.

The photoresist was then developed with 0.85% sodium carbonate solution. The developed micro pattern was used as a mold for obtaining the microchannel. Using soft-lithography techniques^66,67^, polydimethylsiloxane (PDMS) was mixed thoroughly in a 10:1 ratio of silicone elastomer to the curing agent and was degassed in a desiccator. The degassed mixture was then poured onto the mold and was kept in a hot air oven at 65 °C for 2 h for curing. Silane treatment^68^ was performed on the microchannel to reduce the wettability of the channel. For inlets and outlets, holes were punched using blunt needles on the cured PDMS. The final PDMS pattern was bonded to a glass slide using plasma treatment (Cute, Femto Science)**.** Post plasma treatment, the device was placed in an oven for 2 h to enhance the plasma bonding and to retain the hydrophobicity of the microchannel. The dimensions of the microchannel were found to be of 175 µm width and 35 µm height. The fluids were pumped into the device using syringe pumps (Holmarc, India) and the droplet data was collected using a high-speed camera (Photron Fastcam, US).

### Integration of heater to the microfluidic device

The laser ablated polyimide film was bonded to the glass substrate on which the PDMS microchannel was plasma bonded using a double-sided adhesive. The polyimide film was fabricated so as to accommodate the temperature zone with dimensions of 2.5 × 1.5 cm. To ensure proper thermal contacts, the edges of the heater were covered with copper tape and were sealed with silver ink. The integrated device was placed over another plain glass slide leaving space between them for having contact measurement of temperature as shown in Figure S5(a) and a pictorial representation of the integrated device is shown in Figure S5(b). Post integration, voltage was supplied to the heater through the electrical contacts. As mentioned, one of the characteristics of graphene-based heater was rapid heating i.e., quick response. However, the rapid heating of the film was not found to be favorable for this specific application. The reason behind this was that when there was a sudden increase in the temperature, the localized stresses were built up in the glass slide because of which cracks were raised, resulting in the leakage of the fluids passing through the channel (Figure S6). Another effect that was observed at higher temperatures were the Marangoni effect wherein thermal expansion of fluids was observed due to increase in temperature. With increase in temperature i.e., when the temperature was beyond 100 °C, the fluids started expanding and movement in the microchannel was visualized even without any external pumping. The reason behind this being the rise in temperature leading to the decrease in surface tension of the fluid allowing it to expand in the microchannel.

### Synthesis of gold nanoparticles

#### Droplet generation in the microfluidic channel

**Figure 4:**
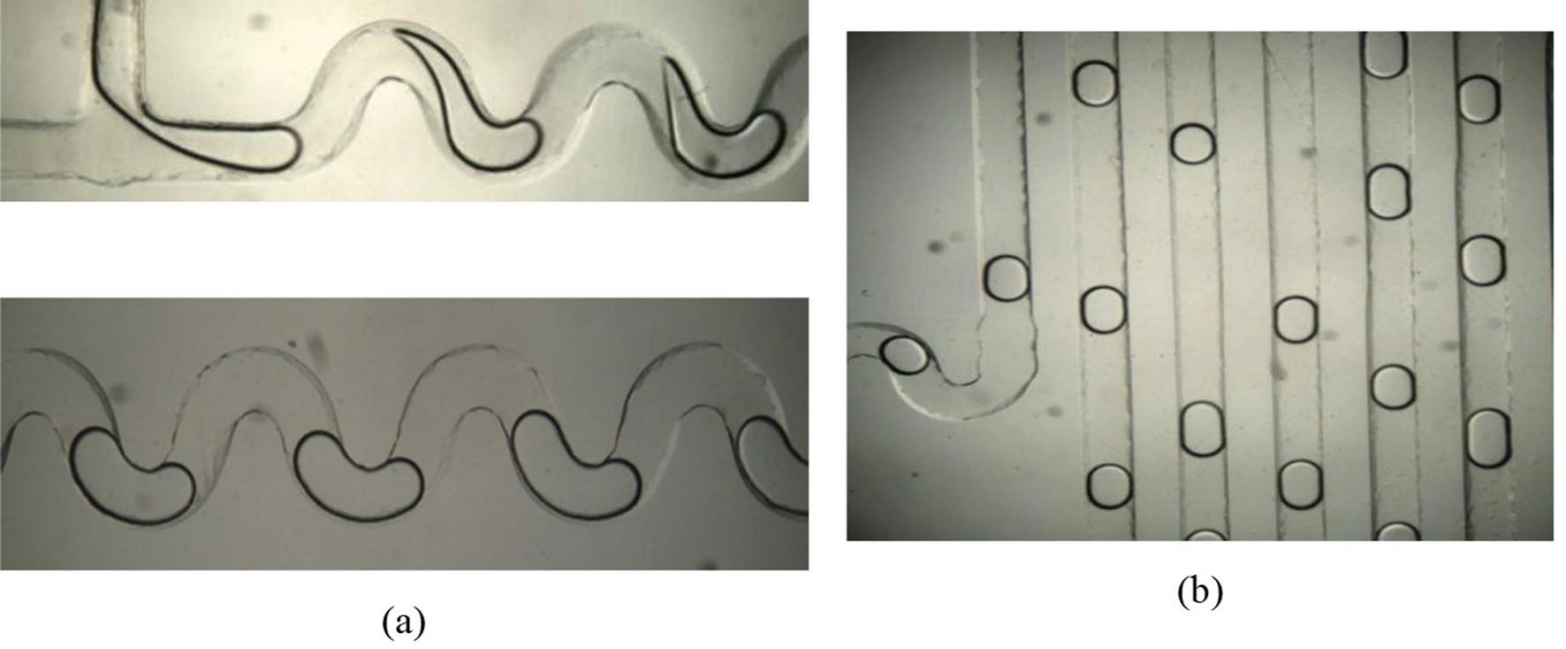
(**a**) Droplet generation and movement in the serpentine channel for a flow rate of 10 µl/min, (**b**) Droplet movement in the heating zone wherein droplets of uniform size can be seen.

Droplet generation in a microfluidic channel is a result of interaction between two immiscible fluids due to the difference in the interfacial tension forces and the viscous forces^69^. The continuous phase shears the disperse phase resulting in the formation of a droplet. In this work, hexadecane was used as a continuous phase and 1% of surfactant (span 80) was used to reduce the interfacial tension and to obtain stable and non-coalescent droplets. The dispersed and continuous phase were passed into the inlets of microchannel. The serpentine channel, provided next to the droplet generation, ensures proper mixing of the contents inside the droplet. The droplet movement in the microchannel can be seen in Fig. 4.

#### Synthesis of gold nanoparticles

The gold nanoparticles are usually synthesized based on Turkevich method wherein the gold(III) chloride solution is reduced by the reducing agent (trisodium citrate) at 90 °C to produce GNP^70^. Following the similar approach, herein two different samples were made for synthesis of nanoparticles using the conventional approach and the microfluidic approach. Initially, 1 mM concentration of HydrogentetraChloroAuarte (HAuCl_4_) (Sigma Aldrich) and 1% of trisodium citrate (Sigma Aldrich) with 38.8 mM concentration were prepared in distilled water^71^. In conventional approach, 10 ml of 1 mM HAuCl_4_ was heated on a magnetic stirrer until it reached a rolling boil. Then, 1 ml of 1% trisodium citrate was added to the boiling solution. The solution was removed from heater once the pale yellow solution of HAuCl_4_ has turned to red.

**Figure 5:**
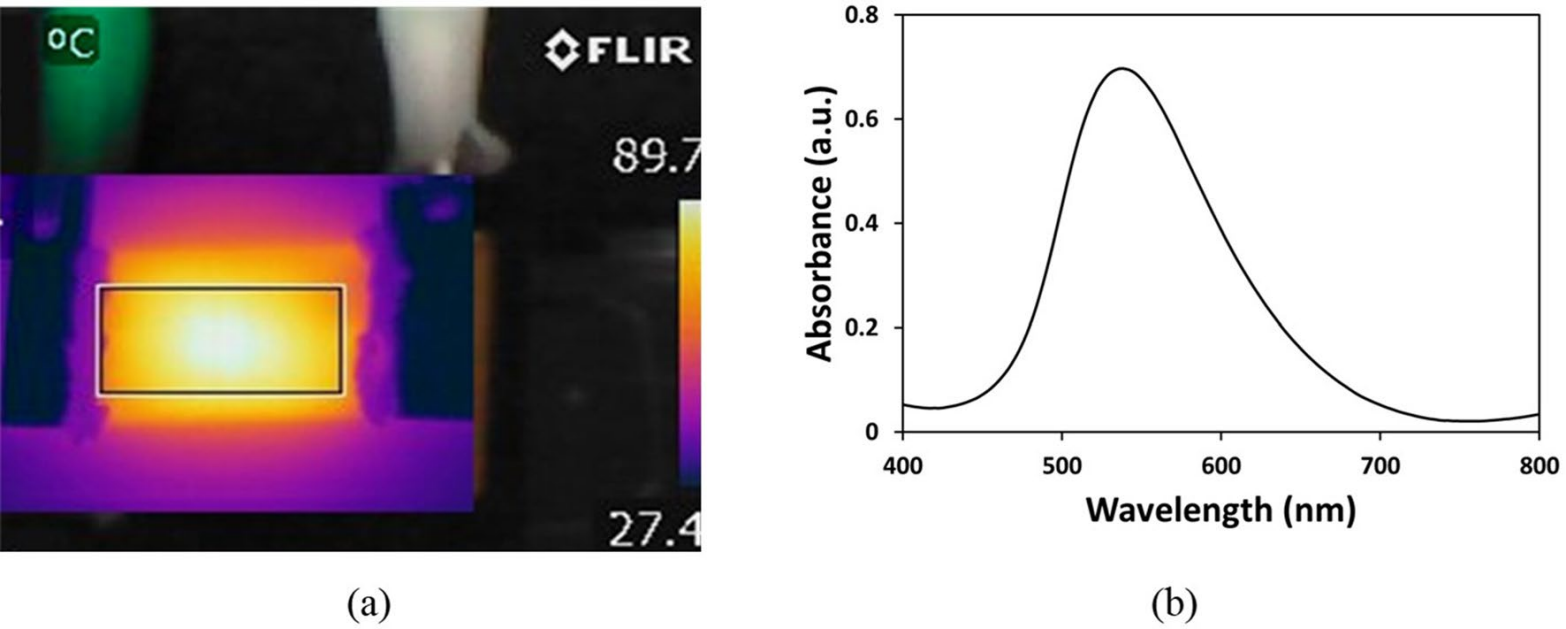
(**a**) Image from thermal camera showing the temperature maintained at 89.7 °C at 6.5 V (**b**) UV visible spectrum obtained for GNP using microfluidic approach.

For synthesis of nanoparticles in droplet based microfluidic platform, 1 mM HAuCl_4_ and 1% trisodium citrate solutions were passed into the microfluidic channel through 2 inlets. This mixture encountered the continuous phase at the T-junction where a droplet was generated. For the mixing of droplet in the microchannel, the droplet passed through the serpentine channel wherein the content in the droplet was mixed because of twirling effect^72,73^. Post mixing, the droplet moved over the zone where temperature was provided with the help of LIG heater. The droplet moved in the microfluidic channel for a time of approximately 30 min. The temperature of the LIG was maintained closer to 90 °C by setting the voltage of 6.5 V for a combination of 10% power and 15% speed as shown in Fig. 5a. The obtained output was then collected in 1.5 ml centrifuge tubes. The tubes were centrifuged at 3000 rpm over a bench top centrifuge to separate the oil from the obtained output.

**Figure 6:**
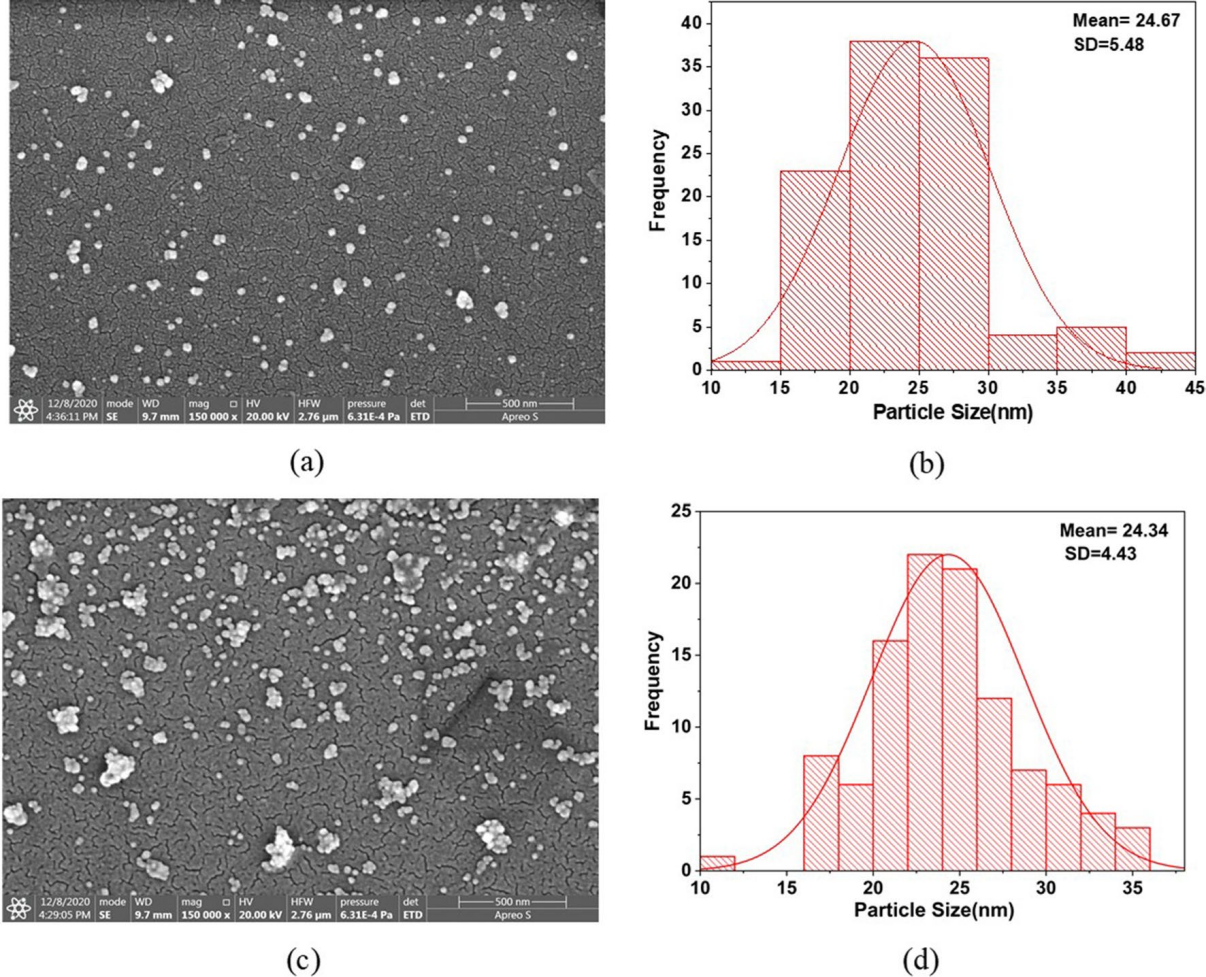
Scanning Electron Microscopy images of GNP with particle distribution obtained through (**a**, **b**) conventional approach (**c**, **d**) Microfluidic approach.

Subsequently, the solution was diluted with DI water and characterization of the solution was carried out through UV Visible (UV–Vis) spectral analysis and FESEM. Figure 5b represents the UV–Vis spectra of the synthesized nanoparticles through the LOC platform. The surface plasmon resonance (SPR) wavelength in the microfluidic approach was found to be 538 nm, which should correspond to the nanoparticle size ~ 20–30 nm as per the earlier work reported on microfluidic platform^63^. Further, in order to identify the size of the particles, FESEM images were acquired (Fig. 6a, c) manifesting that the size of nanoparticles to be almost similar by both the methods (conventional and LOC platform). A mean particle size of 24.67 nm was obtained in the conventional approach whereas it was found to be 24.34 nm through LOC platform. However, as can be seen from Fig. 6b, d that the nanoparticles obtained were found to be monodispersed in conventional approach while polydispersity in the size of nanoparticles was seen in the LOC approach. This could be due to the agglomeration of the synthesized nanoparticles in the microfluidic device because of the presence of minute amounts of oil even after centrifugation. However, with modified microchannel geometry, oil and droplets can be separated at the outlet, and the remaining oil can be removed to obtain aggregate free nanoparticles. This suggests that nanoparticles of much smaller size can be achieved in droplet based microfluidic platform. In short, the fabricated graphene heater serves a key element to provide heating source that can be easily integrated with a LOC platform.

## Functionality and applicability of gold nanoparticles

In order to realize the functionality and applicability of the synthesized nanoparticles, they were employed as catalyst in enzymatic biofuel cell and in electrochemical sensing of analytes such as dopamine and uric acid.

### Enzymatic biofuel cell

The synthesized gold nanoparticles from the LOC platform were used as a catalyst in enzymatic glucose biofuel cell (EBFC). The catalytic property of the GNP was tested in an established platform of paper based microfluidic EBFC with optimized parameters^74^. Similar to the previous reported work from our lab^75^, commercially available Buckypaper (BP) was used as the bioelectrode material and glucose (40 mM) as the fuel. Suitable mediators, such as PBQ (1 mM) and ABTS (1 mM), were used in anolyte and catholyte respectively. The bioelectrodes were prepared by following an established protocol^74^. In brief, 20 µl of GNP solution was pipetted onto the surface of the BP. Then, the electrodes were treated with EDC-NHS (30:90 mM) solution for 1 h. Next, bioanode and biocathode were prepared by dropcasting 20 µl of glucose oxidase (1 mg/ml) and laccase (1 mg/ml) enzyme solutions respectively.

**Figure 7:**
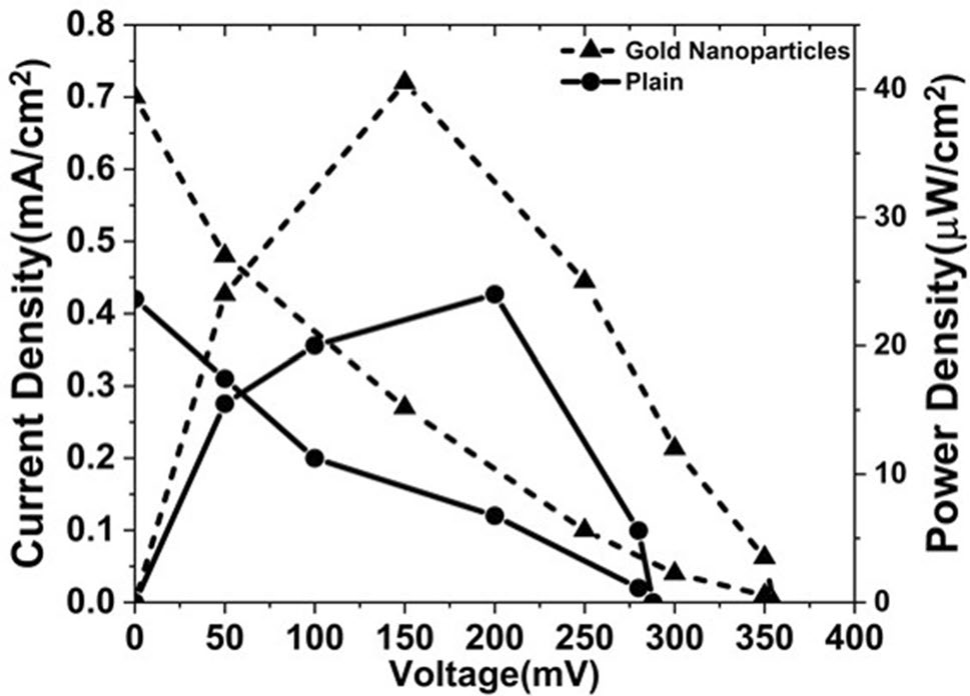
Polarization curves of the enzymatic biofuel cell with bioelectrodes coated with and without GNP.

To observe the effect of GNPs on the performance of EBFC, a control biofuel cell, without GNPs, was created. Figure 7 shows the polarization curves of EBFC integrated with BP bioelectrodes with and without GNPs. The GNP coated EBFC delivered a power density of 40 µW/cm^2^ over a plain EBFC with a power output of 24 µW/cm^2^. The enormous increase (> 66%) in power density is attributed to the high surface area offered by GNPs for efficient immobilization of the enzymes. Also, the catalytic property of GNPs helped in reducing the activation losses of the EBFC thereby increasing its efficiency.

### Electrochemical sensing of Dopamine and Uric Acid

**Figure 8:**
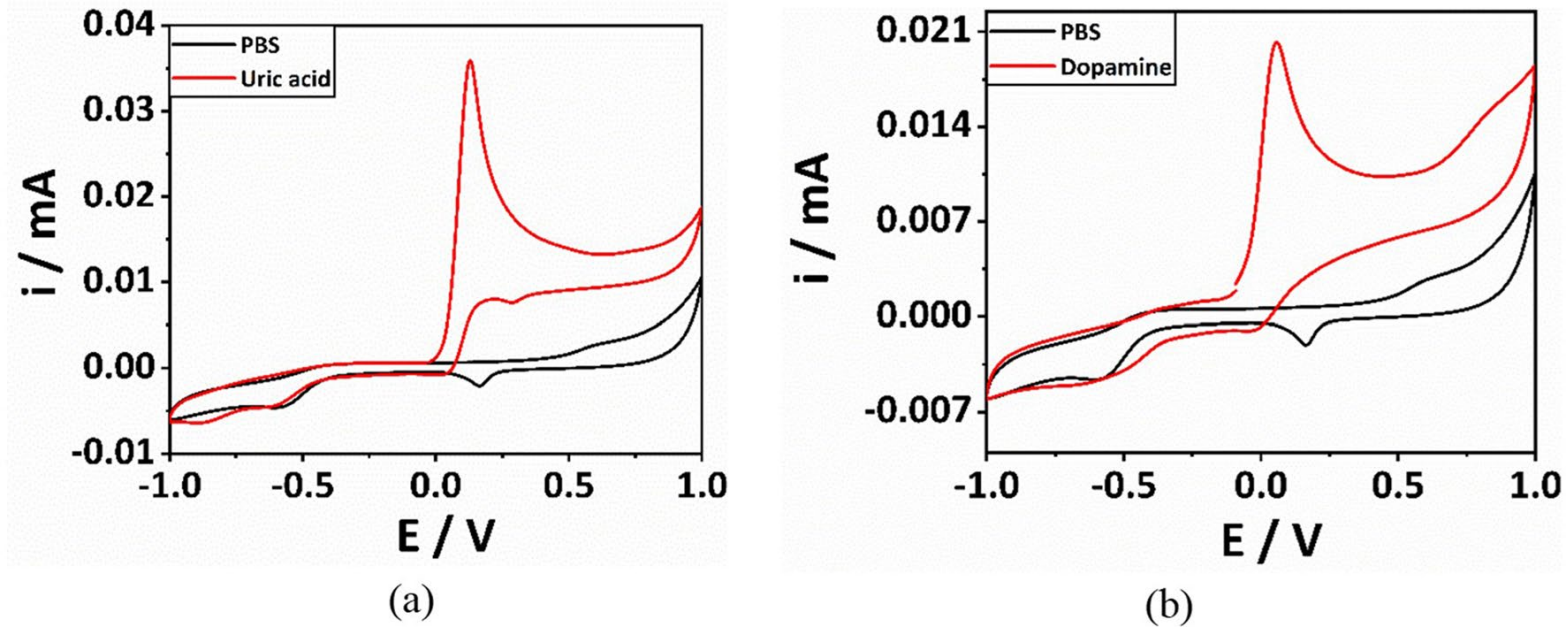
CV response for (**a**) 1 mM Uric acid and PBS and (**b**) 1 mM Dopamine and PBS.

High surface area and the catalytic effect offered by GNPs has led to their usage even in electrochemical detection with high sensitivity^76^. To check the applicability of the synthesized GNPs in a LOC platform, they were utilized for electrochemical sensing of analytes (Dopamine and Uric acid). Initially, 5 μL of GNPs were dropcasted over glassy carbon working electrode (GCE) which was then left to air dry for 12 h at room temperature. After that, the electrode was tested with 1 mM uric acid and 1 mM dopamine in 0.1 M PBS by performing cyclic voltammetry (CV). Figure 8 shows CV obtained for uric acid, dopamine and PBS. In the blank, only the redox peak was observed due to GNPs. Whereas, when tested with uric acid, the oxidation peak was observed at a potential of 0.18 V (Fig. 8(a)) which is in line with the reported work^77^. Subsequently, the electrode was washed with deionized water and tested with dopamine wherein the oxidation peak was observed at a potential of 0.08 V (Fig. 8(b)) nearer to that from the reported work^77^. This clearly suggests that highly sensitive electrochemical sensors can be employed by modifying the electrodes with these synthesized gold nanoparticles that can be used for sensing many biological and chemical compounds.

## Conclusions

Herein, a thin film LIG heater was fabricated and integrated with droplet-based Lab-on-Chip (LOC) platform for various applications. First, the fabricated LIG heater was analyzed at varying combinations of laser power and scanning speed of the laser. The heater showed good stability, quick response and a steady temperature for more than 3 h while maintaining the morphology of the fabricated LIG. Subsequently, the changes in electrical conductivity with varying power and scanning speed of the laser were also demonstrated. The highest and lowest achieved conductivity values were 11.29 $\times$ 10^2^ S/m and 1.97 $\times$ 10^2^ S/m for combinations of 15% power with 5.5% speed and 10% power with 15% speed respectively. A maximum temperature of 589 °C was obtained for combination of 15% power and 5.5% scanning speed. In addition, the fabricated heater was integrated with droplet-based LOC platform. The functionality of the integrated device with LIG heater was demonstrated by synthesizing gold nanoparticles in the droplet-based LOC platform. The synthesized nanoparticles showed excellent behavior as catalysts in enzymatic biofuel cell (EBFC) applications and in electrochemical sensing applications. Overall, the fabricated thin film LIG heater functions as an excellent and cost-effective alternative heating source that can be easily integrated with lab-on-chip applications without compromising any other operation. The presented work has huge relevance in LOC based chemical, biochemical and genomic assays as well as point-of-care (POC) diagnostic applications.

## Materials and methods

### Fabrication of LIG pattern

The patterns were fabricated on Polyimide sheet with a thickness of 10 mil (250 µm) purchased from Dali Electronics. A CO_2_ laser engraving machine (Universal Laser Systems, VLS 3.60) was used to ablate the laser over the polyimide sheet. The maximum power of laser was 30 W and the maximum speed of the scanning stage was 250 mm/s. The ablation process for all the experiments was performed through raster mode over the polyimide sheet. The conductive patterns were fabricated for different combinations of power and scanning speed of the laser. The power of the laser was varied from 2.5 to 15% of the total power of the laser (i.e. 30 W). The scanning speeds were varied from minimum value where carbonization process started to a point until the pattern is ruptured. After fabrication of the conductive films, the edges were sealed with copper tape and covered with silver ink for providing electrical contacts.

### Characterization of the film

Field Emission Scanning Electron Microscope (FESEM) (FEI) images of the conductive film were captured before and after the heating to comprehend the difference. X-Ray photoelectron spectroscopy (Thermo fisher Scientific, K-Alpha) was performed to investigate the chemical structure of the film post ablation of the laser. The electrical conductivity of the ablated patterns was identified using a four-point Probe system from Osilla. The thickness of the film was measured using contact profilometer (Bruker DektakXT).

### Electrical supply and thermal measurements

A portable DC power supply was used for supplying voltage to the fabricated film. The voltage was varied from 6 to 18 V (0.18–0.64 A). Corresponding temperatures were recorded using a thermal imaging camera (Fluke Thermal Imager) and temperature sensor. A PT100 RTD thin film temperature sensor was purchased (Element 14, India) which was coupled to a MAX31865 amplifier to measure the surface temperature of the conductive film.

## Supplementary Information

Supplementary Information
