# Supplementary material for: Droplet-based lab-on-chip platform integrated with laser ablated graphene heaters to synthesize gold nanoparticles for electrochemical sensing and fuel cell applications
Source: Sci Rep. 2021 May 7;11:9750. doi: 10.1038/s41598-021-88068-z (PMC8105317; doi:10.1038/s41598-021-88068-z)
Supplement: Supplementary file 1 — Supplementary Information [file 41598_2021_88068_MOESM1_ESM.pdf]

Droplet-Based Lab-On-Chip Platform Integrated with Laser Ablated Graphene Heaters to Synthesize Gold Nanoparticles for Electrochemical Sensing and Fuel Cell Applications

Sangam Srikanth[1], Sohan Dudala[2], Jayapiriya US[2], J Murali Mohan[1], Sushil Raut[3], Satish Kumar Dubey[1], Idaku Ishii[4], Arshad Javed[1] and Sanket Goel[2*]

[1]Department of Mechanical Engineering, Birla Institute of Technology and Science, Hyderabad 500078, India

[2]MEMS, Microfluidics and Nanoelectronics Laboratory, Department of Electrical and Electronics Engineering, Birla Institute of Technology and Science, Hyderabad 500078, India

[3]Digital Monozukuri (Manufacturing) Education Research Centre, Hiroshima University, Higashi-Hiroshima, Hiroshima, 739-0046, Japan

[4]Smart Robotics Lab, Graduate School of Engineering, Hiroshima University, Higashi-Hiroshima, Hiroshima, 739-8527, Japan

Supplementary Figures

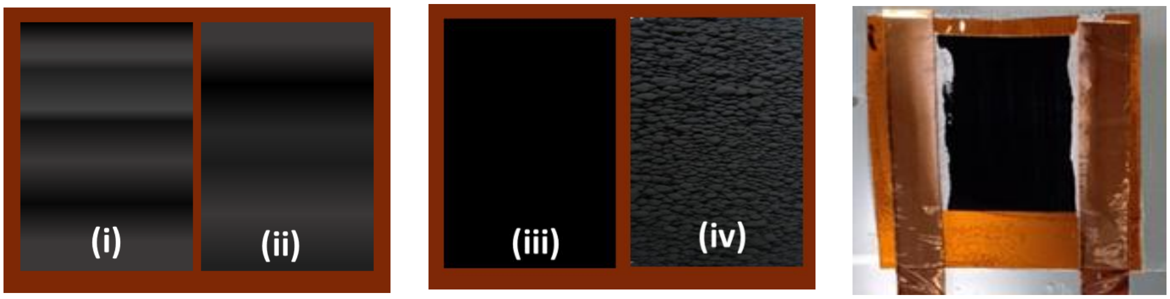

(a) (b)

Figure S1: (a) Pictorial representations of LIG based Carbonization process for different combinations of Laser parameter (i) poor initiation of carbonization at 5% power and 2.5% speed (ii) improved carbonization for combination of 10 % power and 4.5% speed (iii) uniform carbonization of the film at 15% power and 5.5% speed (iv) ruptured film wherein powdered ash was formed at a combination of 15% power and 24% speed **(b)** the conductive film coated with silver ink and sealed with copper tape for electrical contact

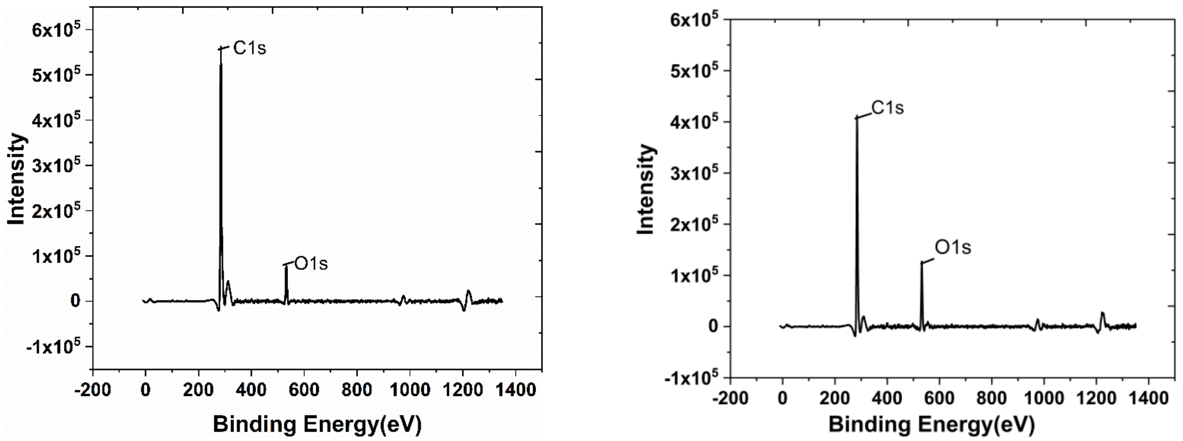

(a) (b)

Figure S2: X-ray photoelectron spectroscopy images of **(a)** Combination of 15% power and 5.5% speed showing 94.1% Carbon and 5.9% oxygen **(b)** Combination of 10% power and 15% speed showing 89% Carbon and 11% oxygen

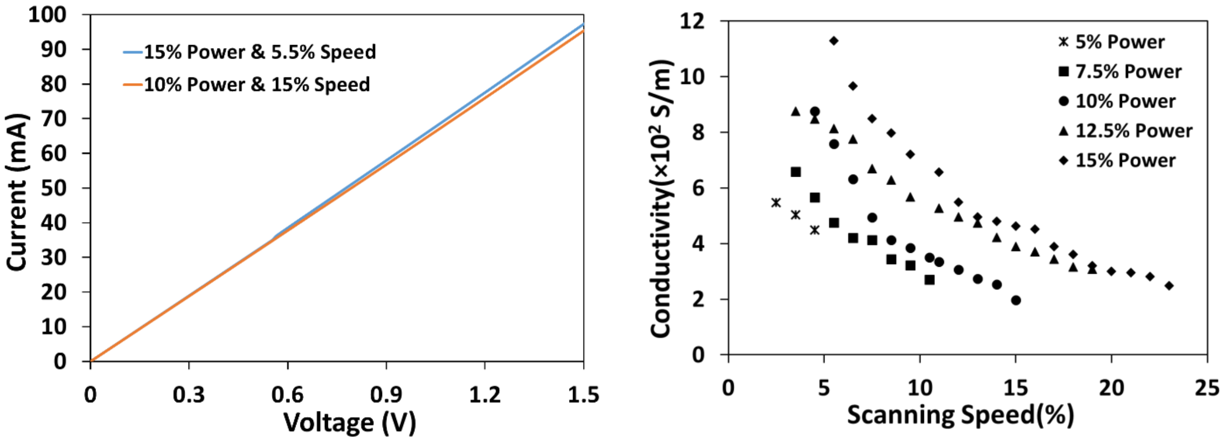

(a) (b)

Figure S3: (a) linear nature of the curve representing uniform conductivity all over the pattern for an applied input voltage **(b)** conductivity values plotted against scanning speed of the laser for different powers of laser

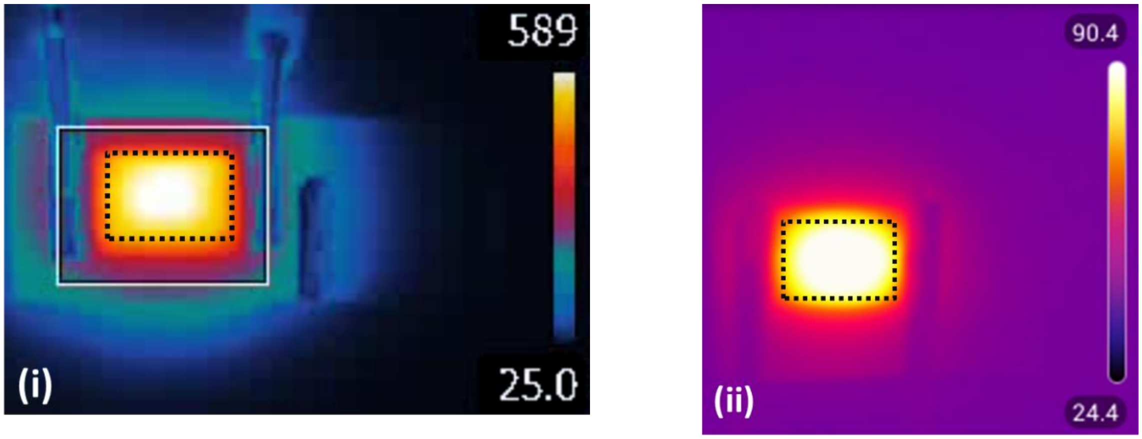

(a)

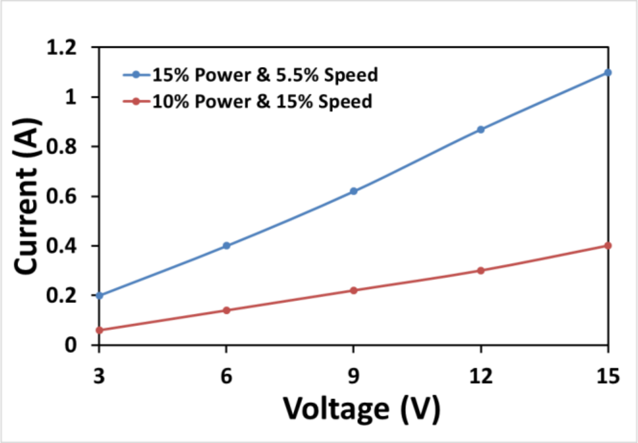

(b)

Figure S4: (a) Effective area of the heater (highlighted in dotted lines) representing **(i)** maximum temperature and **(ii)** the temperature maintained for material synthesis **(b)** Voltage versus current plot representing the amount of current drawn by the heater for a given voltage

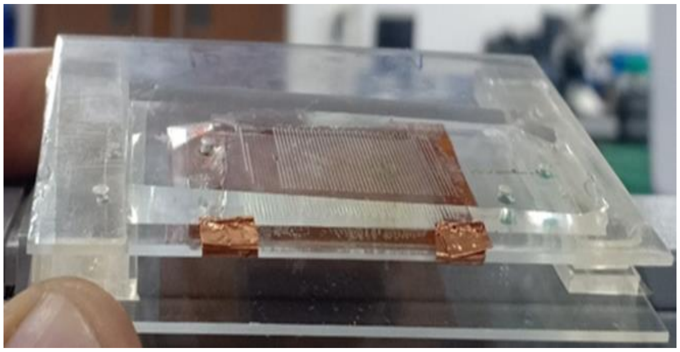

(a)

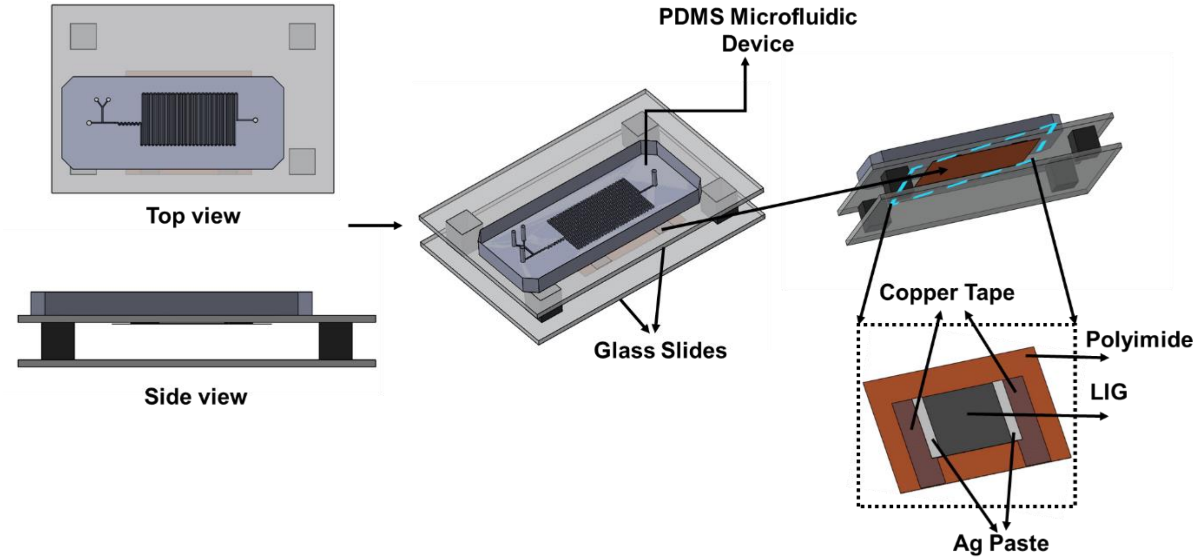

(b)

Figure S5: (a) the final microfluidic chip is placed over another glass slide with provision for measuring temperature of the film using temperature sensor.. Dimensions of the ablated pattern are 1.5×2.5 cm (length × breadth) **(b)** the pictorial representation of the the integrated microfluidic device

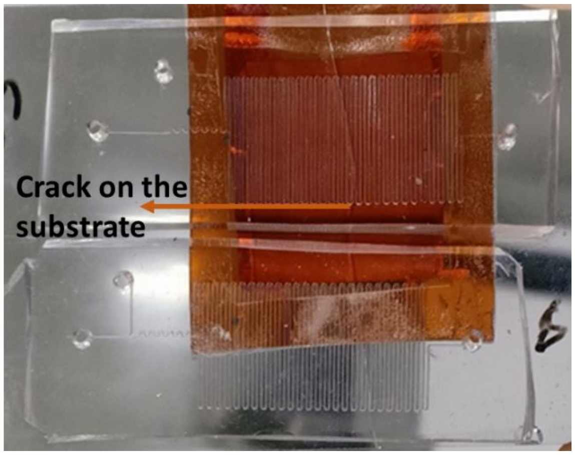

Figure S6: Cracks on the glass slide were formed because of thermal shock due to rapid heating of the graphene heater
